# Impact of Preoperative Cardiac Screening Modality on Cardiovascular Outcomes After Liver Transplantation

**DOI:** 10.1111/ctr.70680

**Published:** 2026-09-15

**Authors:** Giuseppe Esposito, Marcella Manicardi, Niccolò Bonini, Stefano Di Sandro, Marco Vitolo, Fabrizio Di Benedetto, Giuseppe Boriani, Gian Piero Guerrini

**Affiliations:** ^1^ Hepato‐Pancreato‐Biliary Surgery and Liver Transplantation Unit Policlinico Di Modena University of Modena and Reggio Emilia Modena Italy; ^2^ Clinical and Experimental Medicine PhD Program, Department of Surgical, Medical and Dental Department of Morphological Sciences Related to Transplant, Oncology and Regenerative Medicine University of Modena and Reggio Emilia Modena Italy; ^3^ Cardiology Division, Department of Biomedical, Metabolic and Neural Sciences, Policlinico Di Modena University of Modena and Reggio Emilia Modena Italy

**Keywords:** coronary angiography, coronary artery disease, exercise test, heart disease, liver transplantation, tomography x‐ray computed

## Abstract

**Introduction:**

Cardiovascular (CV) disease is a leading cause of morbidity and mortality after liver transplantation (LT). Coronary computed tomography angiography (CCTA) has increasingly replaced stress testing (ST) for CV risk assessment in LT candidates with intermediate‐to‐high CV risk, although comparative evidence remains limited. The primary endpoint was the incidence of post‐LT major adverse cardiovascular events (MACE) by CV screening modality; the secondary endpoint was to identify predictors of MACE.

**Methods:**

This single‐center retrospective study included 506 patients who underwent LT between 2018 and 2023. According to CV screening modality, patients were stratified into three groups: no test (low CV risk), ST, or CCTA.

**Results:**

Among 506 LT recipients, 358 (70.8%) presented low CV risk, 57 (11.3%) underwent ST, and 91 (18.0%) CCTA. Compared with ST, CCTA led to higher rates of coronary angiography (14% vs. 31.9%, *p* = 0.011) and revascularization (0% vs. 15.4%, *p* < 0.0001). After a median follow‐up of 2.5 years [IQR 1.5–3.9], MACE occurred more frequently in CCTA (18.4%) and ST (14%) groups than in low‐risk group (9%) (*p* = 0.041), while no significant difference was observed between CCTA and ST groups (14% vs. 18.4%, *p* = 0.501). In univariate and multivariate Cox‐regression analysis, only age and family history of coronary artery disease were independent predictors of MACE.

**Conclusions:**

CCTA and ST resulted in comparable post‐LT CV outcomes. Although CCTA was associated with a higher number of pre‐LT coronary interventions, it was not associated with a lower incidence of MACE, underscoring the need to refine CV risk stratification protocols in LT candidates.

**Practitioner Points:**

- Currently, there are no international guidelines for cardiovascular screening procedures in LT candidates, leading to variability in practice across different centers and countries. Coronary computed tomography angiography (CCTA) has emerged as a more accurate method than stress testing (ST) in detecting coronary artery disease (CAD) in patients with end‐stage liver disease.
- In the present study, all pre‐transplant coronary revascularizations were performed in patients screened with CCTA, while none occurred in the ST group. Despite this, the incidence of post‐LT major adverse cardiovascular events (MACE) was similar between groups. In our cohort, revascularizations prompted by CCTA did not significantly reduce post‐LT MACE rate. Age and family history of CAD were the only independent predictors of MACE.
- Our results underscore the need to refine cardiovascular risk stratification protocols for LT candidates.

**Abbreviations**
ACS: acute coronary syndrome
AHA: American Heart Association
BMI: body mass index
BPM: beats per minute
CABG: coronary artery bypass grafting
CACS: coronary artery calcium score
CAD: coronary artery disease
CAD‐RADS: coronary artery disease‐Reporting and Data System
CI: confidence interval
CIs: confidence intervals
CCTA: coronary computed tomography angiography
CV: cardiovascular
DAPT: dual antiplatelet therapy
ECG: electrocardiogram
ESLD: end‐stage liver disease
FFR: fractional flow reserve
HCC: hepatocellular carcinoma
HR: hazard ratio
INR: international normalized ratio
iFR: instantaneous wave‐free ratio
LT: liver transplantation
MACE: major adverse cardiovascular events
MASLD: metabolic dysfunction‐associated steatotic liver disease
MELD: Model for End‐Stage Liver Disease
PaO_2_
: partial pressure of arterial oxygen
PTCA: percutaneous transluminal coronary angioplasty
SE: stress echocardiography
ST: stress testing/stress test
TTE: transthoracic echocardiography

## 1 Introduction

Liver transplantation (LT) represents the optimal treatment option for patients with end‐stage liver disease (ESLD) and selective liver tumors. Despite the advancements in surgical techniques and perioperative management, cardiovascular (CV) complications, especially coronary artery disease (CAD), remain a leading cause of morbidity and mortality among LT candidates and recipients, highlighting the importance for thorough preoperative evaluation and cardiac risk assessment [1, 2]. Previous guidelines recommended stress test (ST), such as dobutamine stress echocardiography (DSE) and nuclear myocardial perfusion imaging, to exclude significant CAD in candidates for LT [3]. However, these tests displayed notable limitations in ESLD patients, as hyperdynamic circulatory states and systemic vasodilation can reduce their diagnostic accuracy [4]. In this context, coronary artery calcium score (CACS) and coronary computed tomography angiography (CCTA) have emerged as alternative diagnostic tools [5, 6]. The American Heart Association (AHA) recently recommended CCTA as the preferred method for cardiac screening in LT candidates at intermediate to high CV risk. Although CCTA has shown a high negative predictive value in the general population, its specificity for detecting ischemia‐inducing lesions ranges from 60% to 90% [7, 8]. Whether expanding the use of CCTA for the evaluation of liver transplant candidates leads to improved clinical outcomes, particularly in preventing early and late major adverse cardiovascular events (MACE), remains to be determined.

Moreover, recent evidence, including the ISCHEMIA trial, suggests that routine revascularization does not reduce CV events or mortality compared with optimized medical therapy in asymptomatic patients [9, 10, 11].

The aim of the present study is to assess the incidence of post‐LT MACE between patients screened with CCTA and those screened with ST. By analyzing real‐world data from LT recipients at the Hepato‐Pancreato‐Biliary Surgery and LT Unit—University of Modena and Reggio Emilia—we aim to assess the effectiveness and limitations of our current screening practices and provide a basis for future evidence‐based protocol refinement.

## 2 Materials and Methods

### 2.1 Study Design and Population

This single‐center retrospective observational cohort study was conducted at the Hepato‐Pancreato‐Biliary Surgery and LT Unit—University of Modena and Reggio Emilia. Between January 2018 and December 2023, a total of 654 adult patients were screened for LT; among them, 527 were listed, and 516 ultimately underwent LT. All transplanted patients were consecutively enrolled in the study and stratified into three cohorts based on the type of preoperative cardiac screening test performed according to institutional protocol: baseline cardiac evaluation only (low‐risk cohort), CCTA, or ST. Patients with a history of coronary revascularization, prior LT, or incomplete follow‐up data were excluded.

### 2.2 Cardiac Screening Protocol

**FIGURE 1:**
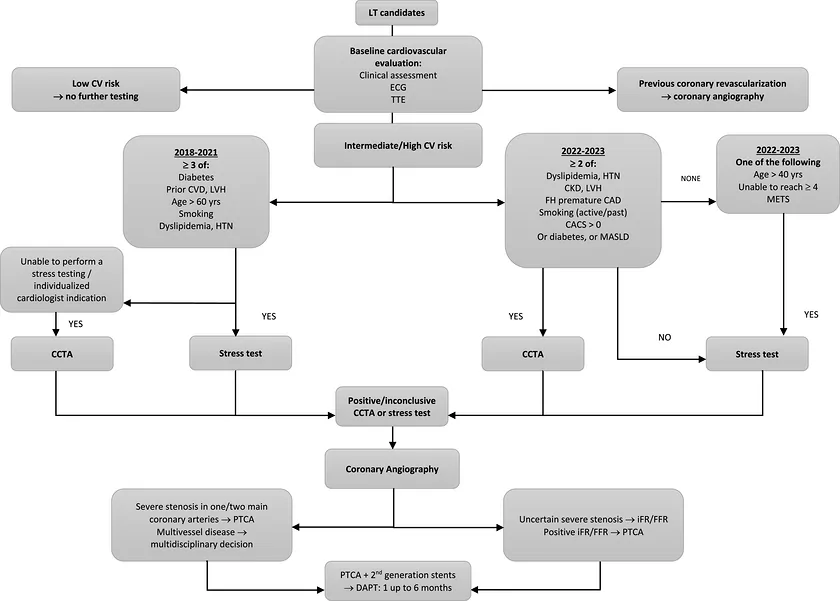
Modena liver transplant protocols for pretransplant cardiac evaluation in LT candidates. CACS = coronary artery calcium score, CAD = coronary artery disease, CCTA = coronary computed tomography angiography, CKD = chronic kidney disease, CV = cardiovascular, CVD = cardio vascular disease, DAPT = dual antiplatelet therapy, ECG = electrocardiogram, FFR = fractional flow reserve, FH = family history, HTN = hypertension, iFR = instantaneous wave‐free ratio, LT = liver transplant, LVH = left ventricular hypertrophy, MASLD = metabolic dysfunction‐associated steatotic liver disease, METS = metabolic equivalents, PTCA = percutaneous transluminal coronary angioplasty, TTE = transthoracic echocardiogram.

Institutional cardiac screening protocols are periodically updated in accordance with evolving evidence and consensus guidelines. According to the 2012 AHA Scientific Statement for CV Screening in LT candidates [3], between 2018 and 2021 patients at intermediate to high CV risk, defined by the presence of at least three of the following risk factors: diabetes mellitus, prior CV disease, left ventricular hypertrophy, age >60 years, smoking, hypertension, or dyslipidemia, underwent noninvasive ST [3]. The choice between a Bruce treadmill exercise test and stress echocardiography (SE) was left to the discretion of the cardiologist. Patients unable to perform an exercise ST, underwent CCTA. In selected cases, CCTA was also performed at the discretion of the consulting cardiologist based on individual patient characteristics. Since 2022, based on the updated recommendations from the 2022 AHA Scientific Statement for CV Screening in LT candidates [12], patients at intermediate to high risk defined by the presence of at least two of the following CAD risk factors: dyslipidemia, hypertension, chronic kidney disease, left ventricular hypertrophy, family history of premature CAD, active or prior tobacco use, or CACS >0, as well as those with diabetes mellitus or metabolic dysfunction‐associated steatotic liver disease (MASLD) have undergone CCTA [12]. Patients aged ≥40 years, those with a history of reduced exercise tolerance (unable to achieve ≥4 METS), as well as those with fewer than three of the aforementioned CAD risk factors, underwent noninvasive stress testing (Figure 1). In the event of inconclusive ST or CCTA results, subsequent investigations were determined by the consulting cardiologist.

Therefore, all patients with CCTA‐detected moderate‐to‐severe coronary stenosis (≥50%), or with a positive ST, subsequently underwent invasive coronary angiography within six months. In instances where the results of CCTA or ST were inconclusive or non‐diagnostic, further evaluation was conducted using either an alternative noninvasive test or coronary angiography, as determined by the consulting cardiologist.

All patients underwent a baseline cardiac evaluation, which included a comprehensive medical history, electrocardiogram (ECG), arterial blood gas analysis, transthoracic echocardiography (TTE), and cardiology consultation. Patients deemed at low CV risk did not undergo additional investigations. Individuals with a history of prior coronary revascularization or ischemic heart disease were directly referred for coronary angiography (Figure 1).

### 2.3 Study Endpoint

The primary endpoint was to determine the incidence of post‐LT MACE, defined as a composite outcome corresponding to the first occurrence of any of the following during follow‐up: myocardial infarction, coronary revascularization (either PTCA or CABG), stroke, CV death, acute heart failure, or clinically significant arrhythmias, in the CCTA and ST cohorts [13].

The secondary endpoint was to identify pre‐LT variables independently associated with post‐LT MACE.

### 2.4 Statistical Analysis

Continuous variables were reported as median and interquartile range (IQR). Comparisons between two groups were conducted using the Mann–Whitney U test, while comparisons between multiple groups were performed using the Kruskal–Wallis ANOVA test. Categorical variables were reported as counts and percentages, and comparisons between groups were evaluated using the Pearson's chi‐squared test. Differences in survival among subgroups with respect to the primary clinical endpoint (composite outcome) were analyzed using the log‐rank test, and survival data were inspected with Kaplan–Meier curves. Pre‐transplant variables associated with the incidence of post‐LT CV events were examined through univariate and multivariate Cox regression models. Results were reported as hazard ratio (HR) with 95% confidence intervals (CIs). A predefined subanalysis including only patients who underwent either CCTA or ST was also performed. A two‐sided p‐value <0.05 was considered statistically significant. All statistical analyses were conducted using IBM SPSS Statistics, version 26 (IBM Corporation, Armonk, NY, USA) and RStudio Version 2025.05.0+496.

### 2.5 Ethics Approval

This study was conducted in accordance with the ethical principles of the Declaration of Helsinki and was approved by the institutional ethics committee (protocol number 622/2024/OSS/AOUMO). All data were pseudoanonymized prior to analysis to ensure the protection of patient confidentiality.

## 3 Results

**FIGURE 2:**
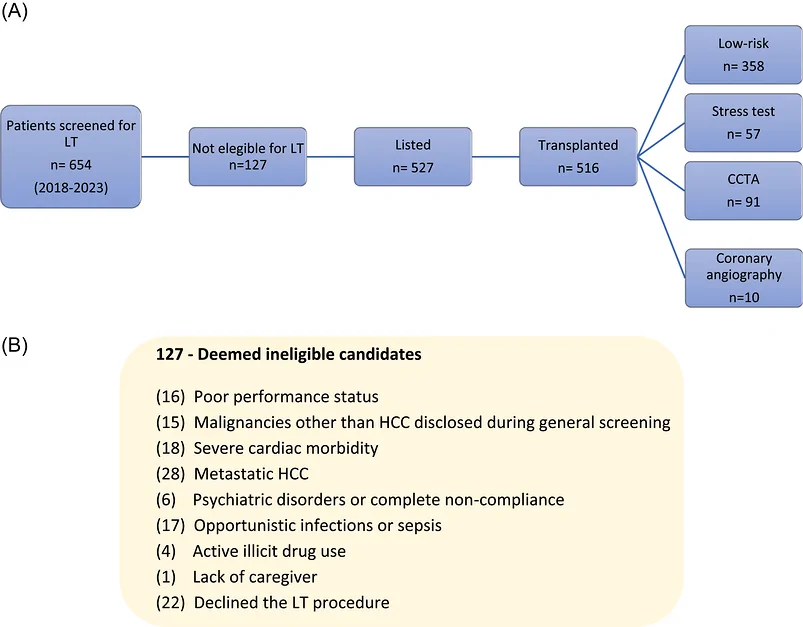
A) Flowchart of the study population. CCTA, coronary computed tomography angiography; LT, liver transplantation. B) Causes of ineligibility for liver transplant. HCC, Hepatocellular Carcinoma

Between January 2018 and December 2023, a total of 654 adult patients were evaluated for LT. Among them, 127 were deemed ineligible for listing, while 527 patients were listed for LT. The median overall wait list time was 86 days [IQR 22–173], and the overall dropout rate was 2.1% (11/527): 10 patients died due to superimposed infections or decompensation of ESLD, and 1 due to progression of HCC. Ultimately, 516 patients underwent LT: 126 received grafts from donation after circulatory death (DCD) donors, 363 from donation after brain death (DBD) donors, and 27 from living donor liver transplantation (LDLT). However, 10 of the 516 transplanted patients had a prior history of coronary revascularization and, in accordance with our institutional CV screening protocols, underwent direct coronary angiography. These patients were excluded from the final analysis (Figures 2A and 2B).

Therefore, a total of 506 liver transplanted recipients were included in the final analysis. The median age was 59 years [IQR 54–65], and 383 subjects (75.7%) were male. The median model for end‐stage liver disease (MELD) score at transplant was 14 [IQR 9–22]. The underlying etiologies of liver disease were as follow: 227 patients (44.9%) had viral hepatitis, including 30 with HIV coinfection; 121 patients (23.9%) had alcohol‐related liver disease; 70 (13.8%) MASLD; and 53 (10.5%) had autoimmune liver disease. Notably, HCC remained a leading indication for LT, accounting for 51.2% (259/506) of all transplanted recipients. Eleven patients (2.2%) underwent LT for primary or secondary liver malignancies other than HCC, and the remaining 24 patients were transplanted for other indications. No difference in waitlist times were observed among the three patient subgroups (low‐risk, ST and CCTA).

Based on CV risk stratification scores, 358 patients (70.8%) were categorized as low risk and underwent baseline cardiac evaluation only (low‐risk cohort), while 57 (11.3%) underwent a ST (ST cohort), and 91 (18.0%) underwent CCTA (CCTA cohort).

Baseline characteristics are shown in Table 1. Patients in the ST and CCTA groups were older (median age 62.5 [IQR 57–67.5] years and 61.5 [IQR 56–66] years, respectively) than those in the low‐risk cohort (58 [IQR 52–64] years, *p* < 0.0001). The median body‐mass index (BMI) resulted also significantly higher in the ST and CCTA groups (26.9 [IQR 24.1–30.0] and 26.4 [IQR 24.2–29.4] kg/m^2^, respectively) compared with the low‐risk group (25.5 [IQR 22.7–28.5] kg/m^2^, *p* = 0.008). The prevalence of CV comorbidities was significantly higher in the ST and CCTA groups: diabetes mellitus (59.6% ST, 60.4% CCTA vs. 11.2% low‐risk, *p* < 0.0001), hypertension (78.9% ST, 62.6% CCTA vs. 27.4%, *p* < 0.0001), dyslipidemia (36.8% ST, 22.0% CCTA vs. 5.9%, *p* < 0.0001), and active smoking (43.9% ST vs. 25.3% CCTA and 27.7% low‐risk, *p* = 0.001). The median MELD score (16 [IQR 10–25.3] vs. 11.5 [IQR 9–14.8] ST and 12.5 [IQR 9–18.3] CCTA, *p* = 0.018), and liver‐function related serum biomarkers (INR, ALT, and total bilirubin) resulted significantly higher in the low‐risk cohort (Table 1).

**TABLE 1:** Baseline characteristics.

	Overall (*n* = 506)	Low‐risk (*n* = 358)	ST (*n* = 57)	CCTA (*n* = 91)	*p* value
**Clinical characteristics**					
Age (yrs)	59 [54–65]	58 [52–64]	62 [57–67.5]	61.5 [56–66]	**< 0.0001**
Male sex	383 (75.7)	257 (71.8)	48 (84.2)	78 (85.7)	**0.002**
BMI (kg/m^2^)	25.8 [23–29]	25.5 [22.7–28.5]	26.9 [24.1–30]	26.4 [24.2–29.4]	**0.008**
MELD	14 [9–22]	16 [10–25.3]	11.5 [9–14.8]	12.5 [9–18.3]	**0.018**
Wait list time (days)	86 [22–173]	83.5 [19–175.5]	79 [29–139]	112 [27–191]	0.332
**Cardiovascular risk factors**					
Hypertension	200 (39.5)	98 (27.4)	45 (78.9)	57 (62.6)	**< 0.0001**
Diabetes	129 (25.5)	40 (11.2)	34 (59.6)	55 (60.4)	**< 0.0001**
Dyslipidemia	62 (12.3)	21 (5.9)	21 (36.8)	20 (22)	**< 0.0001**
Family history of CAD	31 (6.1)	17 (4.7)	6 (10.5)	8 (8.8)	0.120
Smoking status					**0.001**
Active	147 (29.1)	99 (27.7)	25 (43.9)	23 (25.3)	
Former	139 (27.5)	86 (24)	16 (28.1)	37 (40.7)	
Non‐smoker	220 (43.5)	173 (48.3)	16 (28.1)	31 (34.1)	
**Echocardiography**					
LVEF (%)	60 [57–64]	62 [57–65]	59 [55–63]	58.5 [55–63]	**< 0.0001**
EDV (mL)	116 [95–138.3]	115 [86–138]	127 [114–138]	110.5 [89–143.3]	**0.048**
ESV (mL)	46 [37–56]	45 [33–54]	51 [38–59]	45 [36.3–63.3]	**0.019**
Atrial volume (mL)	56 [40–73]	56 [37–74]	60 [49–81]	54 [39–69.5]	0.478
LVH	19 (3.8)	10 (2.8)	4 (7)	5 (5.5)	0.187
**Etiology of liver disease**					0.228
Viremic	227 (44.9)	163 (45.5)	24 (42.1)	40 (43.9)	
MASLD	70 (13.8)	37 (10.3)	12 (21.1)	20 (21.9)	
Non‐HCC liver cancer	11 (2.2)	9 (2.5)	0 (0)	2 (2.2)	
Autoimmune	53 (10.5)	48 (13.4)	3 (5.3)	2 (2.2)	
Alcohol	121 (23.9)	84 (23.4)	13 (22.8)	24 (26.4)	
Other	24 (4.7)	18 (5)	5 (8.8)	1 (1.1)	
HCC	259 (51.2)	170 (47.5)	34 (60.7)	55 (60.4)	**0.012**
**Laboratory tests**					
Hb (g/dL)	11.1 [9.5–13]	11.3 [9.5–12.9]	11.1 [9.5–13.5]	10 [9.2–11.8]	0.856
RBC count (mln/mmc)	3.69 [3–4.36]	3.64 [2.9–4.27]	4.1 [3–4.42]	3.37 [2.64–3.74]	0.378
MCV (fL)	94.3 [88.1–101.3]	94.7 [88.9–102]	93.4 [88.7–101.8]	91.6 [85.9–104.6]	0.334
RDW (cv%)	15.8 [14.5–17.5]	15.7 [14.4–17.8]	16.6 [15.2–17.2]	16.3 [15.5–17.3]	0.859
WBC count (migl/mmc)	4.7 [3.3–6.8]	4.6 [3.3–6.7]	4.9 [3.8–6.5]	4.9 [2.9–5.8]	0.457
PLT (migl/mmc)	82 [52–138.5]	79 [50–119.8]	104.5[62–183.3]	82 [52–130.5]	0.426
INR	1.31 [1.16–1.64]	1.42 [1.2–1.87]	1.3 [1.2–1.56]	1.37 [1.2–1.66]	**0.021**
Creatinine (mg/dL)	0.81 [0.68–1.07]	0.78 [0.67–1.04]	0.75 [0.63–1.17]	0.8 [0.68–1.01]	0.061
eGFR (CG mL/min)	98.6 [71.7–132.7]	97.53 [71.7–133]	111.7 [61.8–145.6]	109.7 [68.5–136.8]	0.389
eGFR (MDRD mL/min)	90.4 [66.4–116.1]	87.8 [70.6–117.4]	100 [62.9–130.4]	92.4 [72.1–120.1]	0.231
Total cholesterol (mg/dL)	131 [93–164]	124.5 [77.3–164]	132.5 [108–167]	140.5 [105.7–152.3]	0.507
LDL (mg/dL)	75 [50.7–100.3]	70.5 [44.3–103.3]	84.5 [57–91.8]	79 [61.8–94.5]	0.221
HDL (mg/dL)	40 [24–53]	41 [20.5–54]	40 [27.3–46]	42.5 [29.5–66.7]	0.444
Triglycerides (mg/dL)	80 [59–118.8]	73.5 [53.8–108.3]	78.5 [59.8–132]	76.5 [61–103.8]	0.137
AST/GOT (U/L)	38 [24–61]	39 [25–61.3]	26.5 [19–39.5]	36.5 [24–48.5]	0.078
ALT/GPT (U/L)	31 [21–52]	31.5 [22–50.5]	24.5 [17.8–28.5]	26.5 [21.8–39.3]	**0.007**
Total bilirubin (mg/dL)	1.72 [0.82–3.79]	2.19 [0.96–5.68]	0.85 [0.59–2.49]	1.56 [0.88–2.63]	**0.001**
Alkaline phosphatase (U/L)	112 [83–160]	113 [81–164.5]	101.5 [89.8–132.5]	115 [81.8–182.5]	0.993
GGT (U/L)	66 [36–128]	63.5 [33.5–110.8]	62 [45.5–196.8]	116 [60–154]	0.086
**Invasive cardiovascular procedures post screening**					
Coronary angiography	37 (7.3)	—	8 (14)	29 (31.9)	**0.011**
PTCA	14 (2.8)	—	0 (0)	14 (15.4)	**< 0.0001**

*Note:* Descriptive data are reported based on available cases; complete information was not available for all participants.

Abbreviations: ALT = alanine aminotransferase, AST = aspartate aminotransferase, BMI = body mass index, CAD = coronary artery disease, CCTA = coronary computed tomography angiography, EDV = end‐diastolic volume, LVEF = left ventricular ejection fraction, LVH = left ventricular hypertrophy, eGFR = estimated glomerular filtration rate, ESV = end‐systolic volume, GGT = gamma‐glutamyl transferase, HCC = hepatocellular carcinoma, HDL = high‐density lipoprotein, INR = international normalized ratio, LDL = low‐density lipoprotein, MASLD = metabolic dysfunction‐associated steatotic liver disease, MCV = mean corpuscular volume, MELD = model for end‐stage liver disease, PLT = platelets, PTCA = percutaneous transluminal coronary angioplasty, RBC = red blood cells, RDW = red cells distribution width, WBC = white blood cells.

Among subjects in the ST cohort, 24 (42.1%) underwent a SE and 33 (57.9%) underwent the treadmill exercise test. ST performances were classified as maximal in 36 (63.2%) cases, submaximal in 17 (29.8%), and nondiagnostic in 4 (7.0%). Eight patients (14.0%) underwent coronary angiography due to positive or inconclusive results; however, none of them required coronary revascularization. In the CCTA group, 26 patients (28.6%) had a CACS >400. Moderate‐to‐severe coronary stenosis was identified in 29 patients (31.9%), who subsequently underwent coronary angiography. Fourteen (15.4%) underwent PTCA. Nine PTCA were performed for single‐vessel CAD, two for two‐vessel involvement, while three patients had multivessel disease. Left anterior descending artery was treated in 12 out of 14 cases, the right coronary artery in 3 cases, and the circumflex artery or its obtuse marginal branch in 5 cases. In all cases, revascularization was achieved through the implantation of second/third generation drug‐eluting stents. Intraprocedural complications occurred in two cases, both involving coronary dissection successfully managed percutaneously. All patients undergoing PTCA received dual antiplatelet therapy with aspirin and clopidogrel. The duration of DAPT was one month in five patients, three months in four, six months in three, and twelve months in one patient. No major bleeding events were reported during follow‐up among patients on dual antiplatelet therapy.

The median overall follow‐up time was 2.5 years [IQR 1.5–3.9]. A total of 41 patients were lost at follow‐up, overall survival was not significantly different among groups (Table 2). During the observation period, 84 patients (18.1%) died. Causes of death included 9 CV events, 17 cases of HCC recurrence, 1 case of acute hepatic artery thrombosis with hemorrhagic shock, 1 suicide, 11 deaths due to invasive pneumonia leading to sepsis, 2 from COVID‐19 pneumonia, and 16 from sepsis‐related complications in the intensive care unit. Additionally, 5 patients died from newly diagnosed malignancies, 3 from recurrent alcoholic cirrhosis, 7 from neurological complications, and 12 died at home due to other or unspecified causes.

**TABLE 2:** Adverse cardiovascular events during follow‐up period.

	Overall (*n* = 465)	Low risk (*n* = 321)	Stress test (*n* = 57)	CCTA (*n* = 87)	*p* value
All‐cause deaths	84 (18.1)	54 (16.8)	9 (15.8)	21 (24.1)	0.158
All‐cause deaths within 90 days post‐LT	27 (5.8)	20 (6.2)	3 (5.3)	4 (4.6)	0.832
CV death	9 (1.9%)	6 (1.9%)	1 (1.8%)	2 (2.3%)	0.822
Stroke/TIA	6 (1.3)	4 (1.2)	1 (1.8)	1 (1.1)	0.992
ACS	7 (1.5)	3 (0.9)	2 (3.5)	2 (2.3)	0.224
PTCA	6 (1.3)	3 (0.9)	2 (3.5)	1 (1.1)	0.596
Arrhythmias	15 (3.2)	4 (1.2)	4 (7)	7 (8)	**< 0.0001**
Acute heart failure	11 (2.4)	5 (1.6)	0 (0)	6 (6.9)	**0.012**
**Composite Outcome (MACE)** ^*^	**53 (11.4)**	**29 (9)**	**8 (14)**	**16 (18.4)**	**0.041** ^**^
					**0.501** ^***^

Abbreviations: ACS, acute coronary syndrome; CV, cardiovascular; PTCA, percutaneous transluminal coronary angioplasty; TIA, transient attack.

^*^Composite Endpoint = CV Death, rePTCA, ACS, Arrhythmias, HF, Stroke.

^**^Composite Endpoint: Chi‐square Test Between Three Groups.

^***^Composite Endpoint: Chi‐square Test Between CCTA and ST, *p* = 0.501.

Adverse CV events observed during the follow‐up period are detailed in Table 2. Stroke or transient ischemic attack (TIA) occurred in six patients (1.3%), with no statistically significant differences between cohorts. Notably, none of these cerebrovascular events were attributed to cardiac arrhythmias. Five events occurred more than three years post‐LT, whereas one was reported 24 days after LT.

Acute coronary syndromes (ACS) were recorded in 7 subjects (1.5%), more frequently in the ST (3.5%) and CCTA (2.3%) groups compared to the low‐risk group (0.9%), with no significant differences among groups (*p* = 0.224). A total of 6 PTCA (1.3%) were performed after LT, more commonly among patients screened with ST (3.5%) and less frequently in the CCTA (1.1%) and low‐risk (0.9%) cohorts (*p* = 0.596). Notably, clinically significant arrhythmias occurred in 8% of patients in the CCTA group and 7% in the ST group, compared to 1.2% in the low‐risk group (*p* < 0.0001). Similarly, the incidence of acute heart failure was significantly higher in these two groups compared to the lower‐risk patients (*p* = 0.012).

**FIGURE 3:**
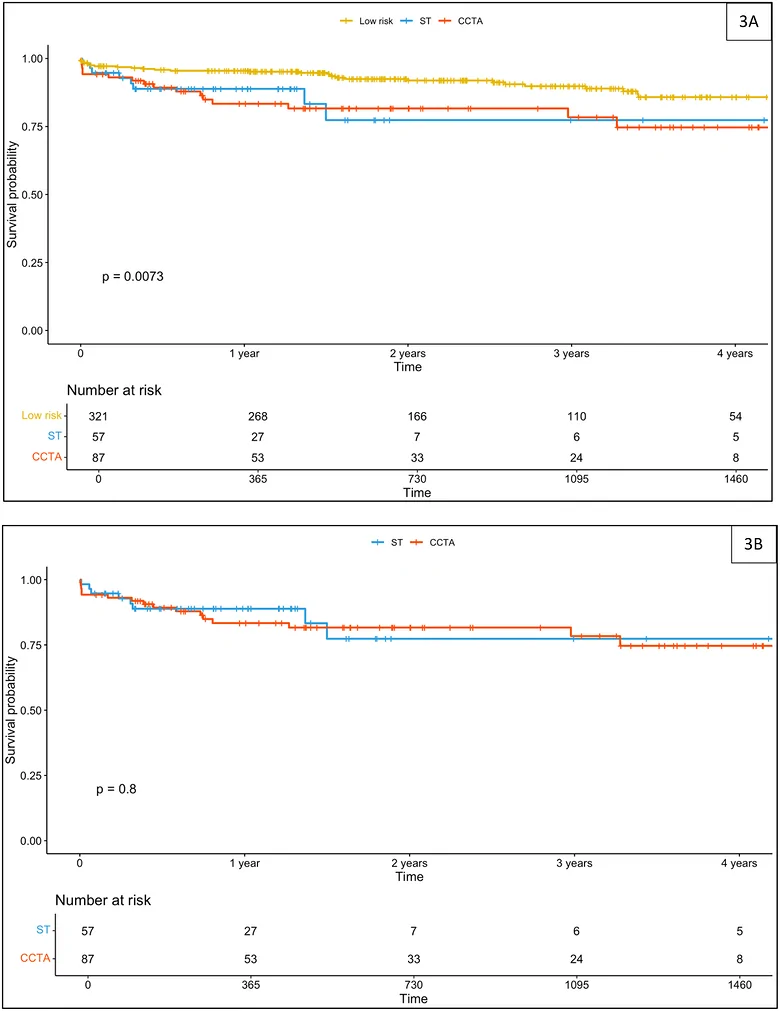
Kaplan–Meier analysis of the incidence MACE after LT. Panel A: comparison among the three study cohorts. Panel B: head‐to‐head comparison between ST and CCTA. CCTA, coronary computed tomography angiography. LT, liver transplantation. MACE, major adverse cardiovascular events. ST, stress test.

The overall survival rates were not significantly different among the three cohorts. The composite outcome (MACE) after LT, occurred in 8 patients (14%) in the ST group, 16 patients (18.4%) in the CCTA group, and 29 (9%) in the low‐risk group (*p* = 0.041). Kaplan–Meier analysis confirmed a significantly lower cumulative incidence of MACE in low‐risk cohort (log‐rank Mantel‐Cox: χ^2^ = 9.848, *p* = 0.0073, Figure 3A). When restricting the analysis to a two‐group comparison between the ST and CCTA cohorts, no significant difference in the cumulative incidence of post‐LT MACE was observed (Log‐rank Mantel‐Cox: χ^2^ = 0.06, *p* = 0.8037, Figure 3B).

In univariate Cox regression analysis, neither CCTA‐based screening nor ST was significantly associated with the occurrence of MACE (CCTA: HR 1.158, 95% CI 0.496–2.701, *p* = 0.735; ST: HR 0.864, 95% CI 0.370–2.015, *p* = 0.735). Similarly, neither CACS >400 on CCTA nor pre‐LT PTCA was associated with a higher risk of MACE (HR = 0.829, 95% CI 0.287–2.398, *p* = 0.729; and HR 2.248, 95% CI 0.843–5.993, *p* = 0.105, respectively). At univariate and multivariate analysis, age and positive family history of CAD emerged as independent predictors of post‐LT MACE (HR 1.128, 95% CI 1.036–1.227, p multi = 0.005; and HR 4.318, 95% CI 1.370–13.610, p multi = 0.013, respectively) (Table 3).

**TABLE 3:** Univariate and multivariate Cox‐regression analysis for the prediction of post‐LT MACE.

	HR (95% CI) *uni*	*p* value *uni*	HR (95% CI) *multi*	*p* value *multi*
Age	1.089 (1.021–1.161)	**0.010**	1.128 (1.036–1.227)	**0.005**
Sex (ref male)	0.737 (0.220–2.463)	0.620	—	—
BMI	1.048 (0.963–1.139)	0.277	—	—
Hypertension	1.103 (0.460–2.644)	0.826	—	—
Diabetes	1.313 (0.580–2.974)	0.513	—	—
Dyslipidemia	1.091 (0.454–2.618)	0.846	—	—
Family history	2.703 (1.011–7.230)	**0.048**	4.318 (1.370–13.610)	**0.013**
Active/previous smoker	0.896 (0.394–2.035)	0.792	—	—
MELD	0.938 (0.875–1.007)	0.076	—	—
HCC	1.664 (0.695–3.984)	0.253	—	—
MASLD	0.700 (0.240–2.041)	0.514	—	—
Alcohol	0.967 (0.386–2.423)	0.943	—	—
Hb pre‐LT	0.988 (0.820–1.190)	0.899	—	—
PLT pre‐LT	1.002 (0.997–1.007)	0.472	—	—
eGFR	1 (0.990–1.011)	0.095	—	—
Delta Hs TnI	2.678 (0.779–9.200)	0.118	—	—
EF	1.012 (0.935–1.095)	0.765	—	—
PTCA post screening	2.248 (0.843–5.993)	0.105	—	—
CCTA	1.158 (0.496–2.701)	0.735	—	—
CACS > 400	0.829 (0.287–2.398)	0.729	—	—
Stress test	0.864 (0.370–2.015)	0.735	—	—

Abbreviations: BMI, body mass index; CCTA, coronary computed tomography angiography; EF, ejection fraction; eGFR, estimated glomerular filtration rate (MDRD); Hb, hemoglobin; HCC, hepatocarcinoma; Hs TnI, high sensitivity troponin I; MASLD, metabolic dysfunction‐associated steatotic liver disease; MELD, model for end‐stage liver disease; PLT, platelets; PTCA, percutaneous transluminal coronary angioplasty.

In the unadjusted Cox regression analysis for MACE, the estimated HR for ST screening compared with CCTA screening was 1.158 (95% CI 0.496–2.701, *p* = 0.735). Even after adjustments (Model 1 adjusted for age, PTCA post screening; Model 2 adjusted for age, PTCA post‐screening, and family history), the type of cardiac screening (CCTA vs. ST) remained not associated with the occurrence of MACE after LT (Table 4).

**TABLE 4:** Risk of post‐LT MACE according to the type of cardiac screening test: Unadjusted and adjusted Cox regression models.

	Unadjusted		Adjusted Model 1		Adjusted Model 2	
	HR (95% CI)	*p* value	HR (95% CI)	*p* value	HR (95% CI)	*p* value
ST	ref	ref	ref	ref	ref	ref
CCTA	1.158 (0.496–2.701)	0.735	1.125 (0.452–2.800)	0.800	1.173 (0.469–2.932)	0.733

*Note:* Model 1 adjusted for: age, PTCA post screening. Model 2 adjusted for: age, PTCA post‐screening, family history.

## 4 Discussion

The primary findings of the present study can be summarized as follows: 1) the choice of pretransplant CAD screening modality (ST vs. CCTA) among intermediate to high CV risk candidates did not impact the incidence of MACE after LT. 2) Patients evaluated with CCTA had a significantly higher prevalence of pre‐LT coronary revascularization procedures (PTCA). 3) Patients stratified as intermediate to high CV risk experienced a higher incidence of post‐LT MACE compared to those at low‐risk.

The 2022 scientific statement from the AHA, endorsed by the American Society of Transplantation, recommends CCTA as the preferred initial test for LT candidates at intermediate to high risk for CAD, introducing a revised definition of intermediate risk [12]. While CCTA has shown to have a high level of sensitivity and a negative predictive value for excluding significant CAD in the general population [14, 15], no direct comparison with coronary angiography in LT candidates is currently available; accordingly, the statement underscores the need for prospective studies to validate the clinical utility and efficacy of this CCTA‐based strategy. In this study, patients with intermediate to high CV risk who were screened with CCTA were more likely to undergo pre‐LT coronary angiography compared to those who underwent ST (31.9% vs. 14%, *p* = 0.011). All pre‐LT PTCA procedures resulting from screening findings were carried out exclusively among the CCTA cohort. There were no periprocedural major hemorrhagic adverse events, nor while on DAPT. However, even after adjusting for coronary revascularization prior to LT, the incidence of MACE did not differ between the ST and CCTA groups (HR 1.125, 95% CI 0.452–2.800, *p* = 0.800, Model 1; HR 1.173, 95% CI 0.469–2.932, *p* = 0.733, Model 2).

Several studies have demonstrated the efficacy of CCTA in the detection of CAD in LT candidates [16, 17]. Nevertheless, the body of evidence is limited to either substantiate or refute the routine practice of pre‐LT revascularization as strategy to reduce post‐LT CV events [12]. Pagano et al. recently disclosed that in a group of 351 LT recipients, percutaneous revascularization before LT was not associated with a different risk of post‐LT CV events, defined as a composite of ACS, arrhythmias, peripheral artery disease and heart failure (HR 0.683, 95% CI 0.076–6.125, *p* = 0.733). Furthermore, among 75 LT recipients who underwent CCTA, a coronary stenosis ≥50% [coronary artery disease‐Reporting and Data System (CAD‐RADS) ≥ 3] at univariate analysis was associated with the incidence of post‐LT CV events (HR 4.455, CI 95% 1.087–19.054, *p* = 0.038). On adjusted analyses, CACS ≥400 and undergoing ST as first‐line CAD screening test remained predictors of post‐LT CV events [8]. Accordingly, in an Australian cohort of 157 transplanted patients who underwent CCTA as a part of pre‐LT cardiac workup, at a median follow‐up of 4 years, CAD‐RADS score ≥ 3 was associated with a heightened risk of MACE, defined as a composite of: myocardial infarction, heart failure, stroke or resuscitated cardiac arrest (HR 5.8, 95% CI 1.6–20.6, *p* = 0.006). However, an increased CACS was not associated with an increased risk of MACE (CACS>100: HR 1.4, 95% CI 0.5–4.4, *p* = 0.98; and CACS>400: HR 2.2, CI 95% 0.6–7.8, *p* = 0.22). Moreover, neither CACS nor CAD‐RAS ≥ 3 were associated with all‐cause mortality [18].

It is important to acknowledged that CCTA alone is not able to provide information on coronary blood flow and has low specificity and low positive predictive value for ischemia‐inducing lesions [19]. One potential approach to overcome these diagnostic shortcomings is the use of CT‐derived fractional flow reserve (FFR) analysis [20]. However, while this approach is feasible in liver transplant candidates, CT‐derived FFR may be less reliable in patients with chronic vasodilation [12]. Further prospective studies are required to determine whether CT‐derived FFR can enhance risk stratification in LT candidates.

Interestingly, within our cohort, the majority of CV events following LT were of a non‐ischemic nature. Arrhythmias occurred in 15 patients (3.2%) post‐LT, while 11 patients (2.4%) developed acute heart failure. In contrast, ACS and PTCA were observed in only 7 (1.5%) and 6 (1.3%) patients, respectively. These findings are consistent with previous literature [21] and may reflect the targeted management strategies implemented for patients diagnosed with CAD during pre‐LT evaluation. However, it is well known that achieving optimal medical therapy for the prevention of CV disease can prove challenging in patients with ESLD. Statins are frequently underprescribed in patients with ESLD [22, 23, 24], despite the recommendation that their utilization should be guided by CV risk rather than lipid levels, as the conventional lipid profile does not reliably reflect CV risk in this subset of population [25]. Aspirin is also frequently underutilized for secondary prevention in LT candidates, despite the lack of evidence supporting a specific platelet threshold as a prerequisite for its safe use in LT [26]. Evidence on the safety of DAPT in patients with ESLD suggests an increased risk of gastrointestinal hemorrhage [27, 28]. Therefore, current recommendations emphasize minimizing DAPT duration, when feasible, and the concomitant prescription of proton pump inhibitors during treatment. Additionally, it remains unclear whether different immunosuppressive regimens may influence the development of adverse CV events following LT [29, 30].

In the present study, 37 patients (7.3%) underwent pre‐LT coronary angiography of whom 14 (2.8%) required percutaneous coronary revascularization. No periprocedural or long‐term hemorrhagic complications were observed. Despite the heightened caution typically exercised in this patient population due to their increased bleeding risk secondary to an altered hemostatic profile, our findings suggest that percutaneous interventions are safe.

The primary goal of pre‐LT cardiac evaluation is to stratify CV risk among candidates by identifying subjects at high risk of perioperative complications, enhancing the assessment of intermediate risks patients, and optimizing medical or interventional strategies to lower long‐term risk of adverse CV events. In recent years, three specific clinical risk scores have been developed to estimate CV risk specifically in LT candidates [1, 31, 32]. However, no formal guidelines have yet been established to standardize preoperative cardiac assessment in LT candidates, and current clinical practice remains largely based on center‐specific protocols. A novel CV risk‐based approach was introduced in the latest AHA Scientific Statement [12], prompting an update of our institutional screening protocol. This study aims to evaluate whether the adoption of the revised protocol for CAD screening has resulted into different post‐LT outcomes.

We found that, among patients at intermediate to high CV risk, the type of cardiac screening, ST versus CCTA, did not significantly impact the incidence of post‐LT composite outcome (MACE). Notably, post‐LT adverse CV events were predominantly characterized by acute heart failure and arrhythmias rather than ischemic events. At our center, adopting a new CCTA‐based protocol led to an increase in number of coronary angioplasties performed pre‐LT during the study period, with no associated hemorrhagic complications during follow‐up.

Although our study is limited by its single‐center retrospective design, we did not observe differences in CV event prevention between patients screened with ST or CCTA, despite a higher rate of coronary revascularization procedures in the CCTA cohort. These findings are in line with data from the ISCHEMIA trial [11], which showed that an initial invasive strategy did not reduce major CV events or mortality compared with optimized medical therapy in patients with stable ischemic heart disease.

This study has several limitations that should be acknowledged. It is a retrospective observational analysis, which inherently carries a risk of selection bias and does not allow for causal inference. Furthermore, the absence of randomization limits the comparability between groups, as the allocation to different screening modalities was based on clinical judgment rather than random assignment. Although the sample size is adequate to detect general trends, it may be underpowered to identify differences in rare outcomes, such as specific subtypes of MACE. Additionally, the exclusion of patients with incomplete records or a history of re‐liver transplantations may have introduced selection bias, potentially limiting the generalizability of the findings. Moreover, the heterogeneity of the included patients, particularly regarding comorbidities, may also have influenced the observed outcomes. Finally, the potential oncological implications of prolonged‐DAPT or exclusions from LT opportunity were not adequately assessed in patients with HCC.

## 5 Conclusion

In our study, all pre‐transplant revascularization procedures were performed in the CCTA group, while none occurred in the ST group. The incidence of MACE was comparable among LT recipients at intermediate to high CV risk who underwent either ST or CCTA as part of their pre‐LT cardiac screening. These findings reflect the greater likelihood of identifying anatomical lesions requiring intervention through CCTA. However, the absence of a significant association between screening modality and MACE suggests that the revascularizations prompted by CCTA did not translate into a measurable benefit in terms of reducing MACE, at least within the limitations of our sample size and follow‐up duration.

Our study highlights the need to refine risk stratification protocols for LT candidates and provides valuable insights into the clinical impact of different preoperative cardiac assessment strategies in this high‐risk population.

## Author Contributions

G.E., MM and G.P.G. conceived the study design, conducted data analysis and interpretation. G.E and M.M curated data acquisition, work drafting, and performed the statistical analysis. F.D.B., G.B., G.P.G., M.V., N.B. and S.D.S critically revised the manuscript for important intellectual content. All authors approved the final version of the manuscript and agree to be accountable for all aspects of the work, ensuring its integrity and accuracy.

## Funding

This study received no funding from any public, commercial, or not‐for‐profit funding agency.

## Conflicts of Interest

The authors declare no conflicts of interest.

## Generative AI Statement

The authors used an artificial intelligence (AI)‐based tool for English language editing. No AI tools were used for data analysis or interpretation. The authors take full responsibility for the content of the manuscript

## Supporting information




**Supporting FIle 1**: ctr70680‐sup‐0001‐SuppMat.docx

## Data Availability

The data that support the findings of this study are available from the corresponding author upon reasonable request.
