# Supplementary material for: Impact of Preoperative Cardiac Screening Modality on Cardiovascular Outcomes After Liver Transplantation
Source: Clin Transplant. 2026 Sep 15;40(9):e70680. doi: 10.1111/ctr.70680 (PMC13575850; doi:10.1111/ctr.70680)
Supplement: Supplementary file 1 [file CTR-40-e70680-s001.docx]

**Diagnostic Procedures**

*Transthoracic Echocardiographic (TTE) evaluation*

All patients underwent preoperative TTE using commercially available ultrasound systems (EPIQ CVx, Philips Healthcare; or Acuson SC 2000, Siemens Healthineers). Left ventricular ejection fraction (LVEF) was derived using the modified biplane Simpson’s method, in accordance to the American Society of [Echocardiography](https://www.sciencedirect.com/topics/medicine-and-dentistry/echocardiography) and European Association of Cardiovascular Imaging [1,2]. In patients with arterial oxygen partial pressure (PaO₂) <80 mmHg, a contrast-enhanced saline bubble echocardiogram was also performed to assess the presence of intrapulmonary or intracardiac shunts.

*Stress Tests*

The stress tests (ST) included either the Bruce Treadmill Exercise Test or Stress Echocardiography (SE), according to the European Association of Cardiovascular Imaging guidelines exercise with semi-supine bike protocol or pharmacological SE were performed [3]. Dipyridamole dose up to 0.86 mg/kg, dobutamine starting from 5 to 40 μg/kg/min with atropine coadministration up to 1 mg, and adenosine up to 0.14 μg/kg/min over 6 min were administered. Throughout the testing, ECG and blood pressure were continuously monitored. The stress tests were interrupted in the occurrence of one or more of the following events: severe chest pain, diagnostic ST-segment abnormalities, excessive blood pressure elevation (defined as systolic pressure ≥240 mmHg or diastolic pressure ≥120 mmHg), limiting dyspnea, achievement of at least 85% of the target heart rate, muscular exhaustion, significant arrhythmias or limiting adverse effects [4].

*Coronary computed tomography angiography (CCTA)*

CCTA scans were acquired using two 128-slice single energy CT scanners (Lightspeed VCT, GE Healthcare). All patients were prepared with intravenous access in the antecubital vein, preferably on the right side. The imaging protocol included two breath-hold acquisitions: a non-contrast ECG-gated prospective axial scan (120 kV, modulated mA, 2.5 mm slice thickness, 250 mm field of view) to assess the coronary artery calcium score and a contrast-enhanced ECG-gated retrospective spiral CT angiography (100–140 kV, modulated mA, 0.6 mm slice thickness, 320 mm field of view). If the resting heart rate exceeded 65 beats per minute (BPM), intravenous metoprolol (5 mg per bolus) was administered to reach a target heart rate of approximately 60 BPM. A triphasic contrast injection protocol was employed: undiluted high-concentration iodine contrast (Iomeron 400, Bracco), followed by a diluted contrast phase (20% iodine / 80% saline), and a final saline flush. The injection velocity was selected to achieve an iodine delivery rate (IDR) between 1.6 and 2.0 grams of iodine per second, with a total contrast volume (typically 50–80 ml) calculated by multiplying the injection rate by the sum of scan delay and scan duration. Bolus tracking was performed with the region of interest (ROI) placed on the ascending aorta.

*Coronary angiography and revascularization*

Coronary angiography was performed in patients with a history of previous coronary revascularization and in patients with significant stenosis on CCTA or positive ST. Significant coronary artery stenosis is defined as ≥50% reduction in caliber of the left main coronary artery or a ≥70% stenosis in any major epicardial vessel [5]. During coronary angiography, percutaneous transluminal coronary angioplasty (PTCA) was performed in the presence of ≥70% stenosis in a main coronary vessel suitable for intervention. In case of multivessel disease (i.e. significant stenosis in all three major coronary vessels [6]), treatment decisions were discussed in a multidisciplinary team meeting. Moreover, the functional assessment using the instantaneous wave-free ratio (iFR) was performed in cases of uncertain stenosis severity. Lesions with iFR values <0.90 were considered suitable for angioplasty. In cases where the iFR value resulted exactly 0.90, additional functional evaluation with fractional flow reserve (FFR) was carried out. When FFR was ≤ 0.80, PTCA was performed accordingly [7]. Second-generation drug-eluting stents were used preferentially to allow shorter dual antiplatelet therapy (DAPT). Following PTCA, patients received a minimum of one month of DAPT. In the absence of bleeding complications and if no high hemorrhagic risk was identified, DAPT was extended to six months, followed by lifelong single antiplatelet therapy.

**Data Collection and Follow-up**

Data were retrospectively collected from institutional electronic medical records and transplant database. Baseline demographic information, medical history, laboratory values, ECG, and TTE parameters were retrieved. Hypertension was identified either as the use of anti-hypertensive medications or as a systolic blood pressure ≥ 140 mmHg or diastolic blood pressure ≥ 90 mmHg measured during outpatient visits or through home monitoring. Diabetes mellitus was defined as either the use of glucose-lowering medications, or a hemoglobin A1_C_ ≥ 6.5%, or a fasting plasma glucose ≥ 116 mg/dL on at least two separate occasions, or a random plasma glucose > 200 mg/dL. Dyslipidemia was defined by treatment with lipid-lowering medications or a total cholesterol ≥ 200 mg/dL. Ischemic heart disease was identified based on a documented history of myocardial infarction or previous coronary revascularization procedures, including coronary artery bypass grafting (CABG) or percutaneous coronary intervention (PCI) with stent placement. Finally, MASLD was diagnosed in patients with hepatic steatosis detected by imaging, serum biomarkers, or histology, in the presence of overweight/obesity, diabetes mellitus, or at least two metabolic risk factors, in accordance with current international consensus definitions [8].

Follow-up was conducted through regular outpatient visits at our transplant clinic. Clinical assessments, laboratory tests, and radiological investigations were performed and documented at each visit, and all relevant data were recorded in the institutional database.

1.	Lang, R. M. *et al.* Recommendations for chamber quantification *. *Eur. J. Echocardiogr.* **7**, 79–108 (2006).

2.	Lohani, O., Colvin, K. L. & Yeager, M. E. Biomarkers for pediatric pulmonary arterial hypertension: challenges and recommendations. *Paediatr. Respir. Rev.* **16**, 225–231 (2015).

3.	Sicari, R. *et al.* Stress echocardiography expert consensus statement: European Association of Echocardiography (EAE) (a registered branch of the ESC). *Eur. J. Echocardiogr. J. Work. Group Echocardiogr. Eur. Soc. Cardiol.* **9**, 415–437 (2008).

4.	Pellikka, P. A. *et al.* Guidelines for Performance, Interpretation, and Application of Stress Echocardiography in Ischemic Heart Disease: From the American Society of Echocardiography. *J. Am. Soc. Echocardiogr. Off. Publ. Am. Soc. Echocardiogr.* **33**, 1-41.e8 (2020).

5.	Knuuti, J. *et al.* 2019 ESC Guidelines for the diagnosis and management of chronic coronary syndromes. *Eur. Heart J.* **41**, 407–477 (2020).

6.	Davies, J. E. *et al.* Use of the Instantaneous Wave-free Ratio or Fractional Flow Reserve in PCI. *N. Engl. J. Med.* **376**, 1824–1834 (2017).

7.	Rinella, M. E. *et al.* A multisociety Delphi consensus statement on new fatty liver disease nomenclature. *Hepatol. Baltim. Md* **78**, 1966–1986 (2023).

8.	Bosco, E., Hsueh, L., McConeghy, K. W., Gravenstein, S. & Saade, E. Major adverse cardiovascular event definitions used in observational analysis of administrative databases: a systematic review. *BMC Med. Res. Methodol.* **21**, 241 (2021).
